# Validity and reliability of the Self-Care Activities Screening Scale (SASS-14) during COVID-19 lockdown

**DOI:** 10.1186/s12955-020-01607-6

**Published:** 2021-01-02

**Authors:** Martín Martínez, Elkin O. Luis, Edwin Yair Oliveros, Pablo Fernández-Berrocal, Ainize Sarrionandia, Marta Vidaurreta, Elena Bermejo-Martins

**Affiliations:** 1 School of Education and Psychology, University of Navarra, Campus Universitario, 31009 Pamplona, Navarra Spain; 2 Institute of Health Research of Navarra (IdiSNA), Pamplona, Spain; 3 School of Psychology, University of San Buenaventura, Bogotá, Colombia; 4 Faculty of Psychology, University of Malaga, Málaga, Spain; 5 Faculty of Psychology, University of the Basque Country, Leioa, Spain; 6 School of Nursing, University of Navarra, Pamplona, Spain

**Keywords:** Construct validation, Self-care screening, Lockdown, COVID-19, Exploratory and confirmatory factor analyses

## Abstract

**Background:**

In a context where there is no treatment for the current COVID-19 virus, the combination of self-care behaviours together with confinement, are strategies to decrease the risk of contagion and remain healthy. However, there are no self-care measures to screen self-care activities in general population and which, could be briefly in a lockdown situation. This research aims to build and validate a psychometric tool to screen self-care activities in general population.

**Methods:**

Firstly, an exploratory factor analysis was performed in a sample of 226 participants to discover the underlying factorial structure and to reduce the number of items in the original tool into a significant pool of items related to self-care. Later a confirmatory factor analyses were performed in a new sample of 261 participants to test for the fit and goodness of factor solutions. Internal validity, reliability, and convergent validity between its score with perceived stress and psychological well-being measures were examined on this sample.

**Results:**

The exploratory analyses suggested a four-factor solution, corresponding to health consciousness, nutrition and physical activity, sleep, and intra-personal and inter-personal coping skills (14 items). Then, the four-factor structure was confirmed as the best model fit for self-care activities. The tool demonstrated good reliability, predictive validity of individuals’ perception of coping with COVID-19 lockdown, and convergent validity with well-being and perceived stress.

**Conclusions:**

This screening tool could be helpful to address future evaluations and interventions to promote healthy behaviours. Likewise, this tool can be targeted to specific population self-care’s needs during a scalable situation.

## Background

Health is defined as “a state of complete physical, social and mental well-being and not merely the absence of disease or infirmity” [1]. However, it can be also considered from a more dynamic perspective as the ability to adapt and self-care in order to face physical, social and emotional challenges [2]. Self-care is considered as an important and valuable principle because it emphasizes the active role of people in maintaining their own wellbeing. Nowadays, there is resurgent interest in the concept and practice of self-care as an essential component of health promotion to improve health, wellness and wellbeing of individuals, and as a strategy to reduce the high costs of medical services [3, 4].

### Psychological impact of COVID-19 lockdown

The current ongoing pandemic coronavirus disease 2019 (COVID-19) has spread around the world while driving global actions. In addition to the fear of contracting the virus, this situation has led to significant changes to our daily lives. In order to support efforts to contain and slow down the spread of the virus, lockdown or mandatory quarantine was globally established. Despite the need of this Public Health recommended measure, our movements are restricted; people are facing new realities of working from home, temporary unemployment, home schooling of children, and lack of physical contact with other family members, friends and colleagues [5]. As result, people are very likely to develop a wide range of symptoms of psychological stress and disorder, including low mood, insomnia, stress, anxiety, anger, irritability, emotional exhaustion, depression and post-traumatic stress symptoms. Low mood and irritability specifically stand out as being very common [6]. However, getting involved in self-care activities as part of hygienic practices can help to manage stress and prevent difficulties and symptoms of health problems [7]. This scenario allows examining the main strategies that people are using for personal self-care, since confinement restrictions entail important changes on their daily habits and routines. This fact leads us to consider that a population brief screening of self-care activities might be used as an important strategy to look for as-yet-unrecognised health risk factors, which later, formal evaluation and intervention strategies can be addressed to.

### Self-care conceptual model

Although the concept of self-care has been broadly used in healthcare literature, many disciplines have provided definitions of self-care from specific perspectives [8–10]. Specifically, Orem’s Self Care Model is the most well-known theory on self-care [11]. This theory identifies two components: the self-care agency (i.e., the ability of a person to engage in self-care) and the self-care behaviours (i.e., the activities performed by a person to maintain life and promote well-being). However, various definitions of self-care have emerged as a result of the lack of consensus, and multiple terms are used as synonyms for self-care, such as, self-agency, self-efficacy, self-management, self-monitoring, and self-help, so it is not always clear how the term is defined [12].

A recent systematic review and concept analysis conducted by Matarese et al. [10] defines self-care as a broad concept that encompasses all the other related concepts which entails capacities, activities, and processes directed toward maintaining health, preserving life, and monitoring and managing acute and chronic conditions. People are supported in this natural process by their self-care abilities (self-care agency); which are prerequisites to care for one’s self, and by self-efficacy; which facilitates the achievement of desired outcomes. Besides, social support is an important part of self-care and people’s family and healthcare professionals are key agents to provide it [10]. Nevertheless, conceptual models neither from academic or lay literature, conceptualize self-care in its totality, nor could explain the link between self-care activities, behaviour change and resource utilisation in the context of the prevailing culture and the external environment.

In an attempt to cover this gap, the Self-Care Matrix (SCM) proposed by El-osta et al. [3] has been proposed as a synthesis of 32 existing conceptual models and frameworks to capture the totality of self-care. This matrix of models includes four cardinal dimensions of self-care that could be addressed and measured separately as a macro, meso or micro-level strategy: (1) Self-Care Activities, (2) Self-Care Behaviours, (3) Self-Care Context, and (4) Self-Care Environment. At micro-level, which this study is aimed to, self-care is considered from a person-centred perspective and covers activities related directly to what individuals can do for themselves, as well as the knowledge required to inform suitable self-care choices. To address this first dimension, the Seven Pillars of Self-Care framework developed by the International Self-Care Foundation has been considered the best candidate to explore the self-care activities dimension [3]. This model involves seven personal activities such as, knowledge, self-awareness and health literacy, psychological well-being, physical activity, healthy eating, good hygiene and the avoidance of risks such as, tobacco and excessive alcohol consumption and rational use of products and services.

Moreover, self-care activities are directly related to the health consciousness concept, which refers to self-awareness about one’s health, and the willingness to engage in health and wellness promoting behaviours [13–15]. In this way, self-care activities are a predictor of well-being, which may be determinant of wellness participation [15]. It leads health conscious individuals to actively seek information about how to improve their health, and adhere to health behaviours accordingly [16–18]. Hence, individuals with high health consciousness have positive attitudes about self-care activities and have healthier lifestyles than individuals with low health consciousness [13, 19, 20].

### Self-care measures

Several instruments have been developed to assess self-care in many different populations, and for various health conditions, such as patients with type 2 diabetes, [21], people with hypertension [22], children [23], or general adult population [24].

A recent systematic review was specifically conducted on the instruments designed to assess self-care in health promotion and maintenance in the adult population [25] in which nine instruments were identified: Appraisal of Self-care Agency Scale (ASA-A) [26, 27], Denyes Self-Care Practice Instrument (DSCPI-90) [28], Denyes Self-Care Agency Instrument (DSCAI) [29], Exercise of Self-Care Agency (ESCA) [30], Lorensen’s Self-care Capability Scale (LSCS) [31], Perceived Self-Care Agency Questionnaire (PSCAQ) [32], Self-care Ability Scale for the Elderly (SASE) [33], Self-as-Carer Inventory (SCI) [34], Self-Care of Home-Dwelling Elderly (SCHDE) [35]. In the above-mentioned revision most of the selected studies presented methodological limitations and their quality was rated as “negative or indeterminate” [25].

Despite the number of developed instruments related to the self-care concept, most of them are mainly based on the self-care agency attribute, which is defined as the capabilities of an individual to recognize his or her own needs and to assess personal and environmental resources [36]. However, to our knowledge, none has been identified to screen, assess or evaluate the specific dimension of self-care activities considering health consciousness as a key element of self-care.

Therefore, there is a need to develop a brief screening tool with appropriate psychometric properties (reliability and validity), to take measures to evaluate the self-care activities, including health consciousness dimension and that can be applied in similar situations when health and well-being is compromised. In this way, the focus of this study is to build a valid and reliable short tool for screening self-care activities in Spanish-speaker population during COVID-19 lockdown.

The specific objectives covered in this study were: (1) to design and explore the factorial structure of an original set of items to screen self-care activities in a COVID-19 confined sample, and (2) to confirm the factor structure of the tool in an independent COVID-19 confined sample. In addition, we examine the reliability of the proposed scale and its convergent validity with well-being and stress measures.

## Methods

### Participants

In this study, two samples of general population from Colombia were recruited through an online survey spread through social media. Participants were randomized to select a minimum of 30 people within the following five age ranges: 18–28, 29–39, 40–49, 50–59, and older than 60. As result, Sample 1 (i.e., exploratory sample) was composed 226 participants, whereas sample 2 (i.e., confirmatory sample) consisted of 261 participants. Individuals’ information of both samples was collected at the same time, specifically, during the beginning of the COVID-19 lockdown (i.e., from 31th March to 14th April of 2020). Ethical approval was obtained by the Research Ethics Committee of the University of Navarra (Project ID: 2020.058) and by the Colombian standards for research in psychology.

### Procedure

First, socio-demographic data and items related to COVID-19 lockdown were included (i.e., age, sex, city, country, socio-economic status, level of studies completed, professional group, being in charge of older and children), employment situation previous and subsequent to COVID-19 lockdown, information related to COVID-19 lockdown (i.e., number of days in confinement, number of people living with and health status (i.e., historical psychological and physical illnesses).

In order to create the initial pool of items related to self-care, authors reviewed the literature for existing scales attempting to assess self-care. Based on the Seven Pillars of Self-Care framework [3], its self-care activities dimensions were operationalized. Nevertheless, some of the framework’s activities were merged in one unique dimension as well as, others important self-care activities were newly included to complete this model. After this conceptual process, two authors: EB (nurse specialized in the theoretical field of self-care, and EL (psychologist specialist in the operationalization of psychological constructs) developed 17 items each, resulting in 34 items covering these activities. As result, 9 self-care activities dimensions: 1. Health Consciousness (6 items); 2. Intrapersonal Skills (2 items); 3. Social Support (3 items); 4. Physical Activity and Healthy Eating (4 items); 5. Sleep quality (2 items); 6. Spare Time activities (3 items); 7. Hygiene (2 items); 8. Information Attitude Consumption (4 items), and 9. Substance Abuse (8 items). It is worthy to mention that items from the health consciousness dimension were translated and adapted from the Health Consciousness Scale (HCS) [15] available in English language.

From the initial 34 items, we removed those with binary responses, lasting a total of 24 items which were organized by dimensions and coded in Likert scale ranging from 1 = Never to 6 = Always depending on each self-care activity frequency. The instrument was then uploaded on an online platform to be self-administrated.

### Statistical analyses

#### Construct validity

Descriptive statistics and reliability and validity analyses were conducted using Stata 15. In order to achieve a psychometrically sound measure that holds under validation, exploratory factor analysis (EFA) was used as a first step over sample 1, whereas confirmatory factor analyses (CFAs) were applied later in an independent sample (i.e., sample 2). The first goal was to reduce the set of 24 items to those that best exemplified the proposed dimensions without loading too high on one or more of the other dimensions. EFA can be used to determine whether the hypothesized factor structure is actually reflected in the collected data, and allows reducing the number of items to keep those with the strongest indications of conforming to the proposed underlying structure [37]. In contrast to the EFA, CFA provides a more restrictive test of the hypothesized factor structure by permitting imposed restrictions on relationships between observed variables and factors [38].

#### Exploratory factor analysis

The internal consistency of the initial 24 items was estimated through the Cronbach’s alpha coefficient measured over sample 1. First, we checked the conditions for a stable factor structure in the data through the Kaiser–Meyer–Olkin measure and we checked for overall significance in the correlations within the items’ correlation matrix by means of a Bartlett’s test of sphericity. An EFA with principal component analysis (i.e., to allow for finding linear combinations of the variables with the greatest variance) was employed to extract the latent dimensions of the original questionnaire, where an orthogonal Varimax rotation (i.e., to minimize the number of variables with high loading on each factor, and to simplify the interpretation of the factor solution) was selected. We retained those items with factor loadings greater than 0.5 and with a minimum difference in factor loading on the remaining factors of 0.2 [39], items that would compose the final scale. Determining the number of factors of the final solution in the exploratory sample was guided by parallel analysis with 500 randomly correlated matrices [40]. With parallel analysis a random generated set of Eigenvalues is compared to the empirically derived Eigenvalues.

#### Confirmatory factor analyses

We used CFAs so that the hypothesized factor solution obtained during EFA in sample 1 can be tested for its fit to the observed covariance structure in an independent sample. According to recommendations for scale development [41], CFAs within the structural equation modelling framework were applied to test the underlying factor structure of the solution obtained during EFA in sample 2. All tests were conducted using maximum-likelihood estimation with a logit link function to account for the ordinal nature of the response scale. CFA models with one and n-correlated factors were considered since a unidimensional solution was initially hypothesized, but we also considered the n-factor solution resulted in the EFA. In the first CFA, we analysed the fit of a uni-factorial model with the solution determined by the EFA. In the second CFA, we analysed the fit of a correlated n-factor model with the solution determined by the EFA. To indicate goodness of fit for the model, we used the chi-square measure, Comparative Fit Index (CFI), Tuker Lewis Index (TLI), Standardized Root Mean Squared Residual (SRMR) fit index, Bayesian information criterion (BIC), Akaike’s information criterion (AIC), and Root Mean Square Error of Approximation (RMSEA). Good fit is indicated by values under 0.06 for RMSEA, values above 0.90 for CFI, and values close to 0.95 for TLI [42]. The internal consistency of the scale and its subdimensions was measured by means of the Cronbach alpha’s coefficient.

### Convergent validity

In addition to self-care, the Spanish adaptation of the Ryff’s Psychological Well-Being Scale-29 (PWBS-29) [43] and the Perceived Stress Scale (PSS-10) [44] were administered in order to evaluate for convergent validity in the self-care’s tool. The internal consistency of the PWBS-29 and the PSS-10 was evaluated by means of the Cronbach alpha’s coefficient.

The PWBS-29 is composed of 29 items scaled from 1 to 6 and structured in six dimensions, namely: Self-acceptance (SA), Positive relationships with others (PRO), Autonomy (ATM), Environmental mastery (EM), Purpose in life (PL), and Personal growth (PG), with a minimum score of 29 and a maximum score of 174. The Spanish version of the PWBS-29 has adequate psychometric properties [43] with acceptable to high internal consistency in its subscales (from 0.68 to 0.83).

The PSS-10 is a short questionnaire composed of 10 items (from 0 = Never to 4 = Very often) that evaluates the perceived stress during the last month. The Spanish version of the PSS-10 has adequate psychometric properties with high internal consistency (α = 0.81), and acceptable test–retest reliability (r = 0.73) [45].

Considering the relationship between self-care and well-being [46] and with perceived stress [47], we expected significant positive and negative correlations between their scores, respectively. A value of *p* < 0.05 was selected as a significance threshold, whereas Bonferroni correction method was applied to correct for multiple comparisons.

## Results

### Participants

Participants of sample 1 (i.e., exploratory sample) ranged from 19 to 80 years old (M = 38.31, SD = 12.40), and consisted mainly of females (54.9%, 124/226). In relation with the education level, the majority of the participants (73.4%, 95/166) had finished university education, whereas the 13.7% had finished high school (12/31), 11.9% had finished technical studies (16/27), and 1% had finished elementary education (1/2). The economic monthly income of the sample was as follows: the 17.5% had no income (27/40), the 11.9% earned less than a minimum wage monthly (mwm = 300 USD) (16/27), the 16.4% earned 2 mwm (18/37), the 18.6% earned 3 mwm (22/42), the 12.4% earned 4 mwm (12/28), the 15% earned more than 5 mwm (16/34), and the 18.8% preferred not to answer (13/18).

Participants of sample 2 (i.e., confirmatory sample) ranged from 19 to 90 years old (M = 44.36, SD = 16.11), and consisted of mainly female (61.3%, 160/261). In relation with the education level, the majority of the participants had finished university education (73.95%, 121/193), whereas the 11.1% had finished high school (13/29), 14.2% had finished technical studies (25/37), and 1% had finished elementary education (1/2). The economic monthly income of the sample was as follows: the 13.4% had no income (25/35), the 9.6% earned less than 1 mwm = 300 USD (18/25), the 13.4% earned 2 mwm (24/35), the 21.5% earned 3 mwm (36/56), the 9.6% earned 4 mwm (15/25), the 21.5% earned more than 5 mwm (23/56), and the 11.1% preferred not to answer (19/29).

### Exploratory factor analysis

The internal consistency of the 24 items was α = 0.807. The Kaiser–Meyer–Olkin measure for sampling adequacy exhibited high strength in the relationships among items (KMO = 0.821). The Bartlett’s test of sphericity demonstrated significance in all the correlations within the items’ correlation matrix (χ^2^ = 1718.56, *p* < 0.001). Thus, both tests indicated that the present data were appropriate to use on a factor analytic model.

The EFA was performed with all 24 items over the exploratory sample 1. During factor extraction, eight factors were found with the following Eigenvalues: 3.64, 2.43, 2.29, 2.22, 1.55, 1.23, 1.21, and 1.15. Nevertheless, parallel analysis indicated that only four factors should be extracted. Therefore, we may conclude that four factors probably are the most accurate number to be extracted from these data. The four-factor solution obtained in the EFA explained 43.3% of the total variance in the data matrix with 14 items loading on this solution, whereas the analysis discarded the other 10 items. Thus, the items that best fitted the four factors mentioned above were selected to compose the Self-Care Activities Screening Scale (SASS-14). These items and their factor loadings and unique variances after rotation are indicated in Table 1.

**Table 1:** Rotated solution generated on the EFA in Study 1 (n = 226)

Item number	Item content (Spanish)	Item content (translated to English)	Factor 1	Factor 2	Factor 3		Factor 4	Uniqueness
*SASS*_*1*_	*Estoy alerta de los cambios en mi salud*	*I am alert to changes in my health*	0.77					0.30
*SASS*_*2*_	*Por lo general, soy muy consciente de mi salud*	*I am usually aware of my health*	0.73					0.33
*SASS*_*3*_	*Reflexiono mucho sobre mi salud*	*I reflect about my health a lot*	0.86					0.23
*SASS*_*4*_	*Estoy atenta/o a mis sentimientos con respecto a mi salud*	*I know my inner feelings about my health*	0.80					0.27
*SASS*_*5*_	*Estoy constantemente examinando mi salud*	*I am constantly examining my health*	0.76					0.30
*SASS*_*6*_	*Realizo actividad física (algún deporte, yoga o baile) durante al menos 30 minutos diarios*	*I do physical activity (some sport, yoga or dance) for at least 30 min a day*		0.75				0.25
*SASS*_*7*_	*Como tres porciones de fruta y dos de verdura diariamente*	*I eat three servings of fruit and two of vegetables daily*		0.50				0.36
*SASS*_*8*_	*Considero que estoy comiendo mejor que antes (menos azúcar, sal, aperitivos fritos o comida precocinada)*	*I think I am eating better than I used to (less sugar, salt, fried snacks or pre-cooked food)*		0.51				0.46
*SASS*_*9*_	*Estoy bebiendo una media de 8 vasos de agua diarios*	*I’m drinking an average of 8 glasses of water a day*		0.79				0.34
*SASS*_*10*_	*Duermo entre 7–8 horas diarias*	*I sleep 7–8 h a day*				*0.86*		0.23
*SASS*_*11*_	*Considero que mi descanso es de calidad*	*I think that my rest is of quality*				*0.83*		0.26
*SASS*_*12*_	*Estoy aprendiendo a hacer cosas nuevas como: tocar un instrumento, deporte, practicar un idioma, cocinar, pintar, nuevas apps, videojuegos, *etc.*…*	*I am learning to do new things like: playing an instrument, sports, practicing a new language, cooking, painting, new apps, video games, *etc. *…*					*0.51*	0.43
*SASS*_*13*_	*Participo activamente en las iniciativas de mi comunidad (p.ej: aplaudir, cantar, poner música, ofrecer mi apoyo en lo que pudiera ayudar, *etc*.)*	*I actively participate in the initiatives of my community (eg: clapping, singing, playing music, offering my support in what I could help, *etc*.)*					*0.70*	0.31
*SASS*_*14*_	*Estoy encontrando momentos para estar más conectada/o conmigo misma/o (observo, escribo o reflexiono sobre mis pensamientos, emociones o conductas)*	*I am finding moments to be more connected to myself (I observe, write or reflect on my thoughts, emotions or behaviors)*					*0.71*	0.34

The values of the factors’ variances after rotation were 3.19 (33.9%) for Factor 1, 2.10 (22.3%) for Factor 2, 1.57 (16.7%) for Factor 3, and 1.16 (12.3%) for Factor 4, that explained the 85% of the total variance in the model, with a total of 14 items. Based on these results, three dimensions kept the initial content and label, (1) Health consciousness (HC, 5 items), (2) Nutrition and Physical Activity (NPA, 3 items), (3) Sleep quality (SLP, 2 items), whereas the fourth dimension was composed of 4 items from the original Social Support, Intrapersonal Skills and Spare Time Activities dimensions, and was labelled as (4) Interpersonal and Intrapersonal coping strategies (IICS).

It should be noted that a comparison of the original orthogonal Varimax rotated solution with the oblique Oblimin solution showed that both results were comparable, lending support to this item selection. The reliability of the SASS-14 was α = 0.831, demonstrating good internal consistency in sample 1. The 14 items of the proposed SASS-14 were subjected to CFAs on an independent sample.

### Confirmatory factor analyses

**Fig. 1:**
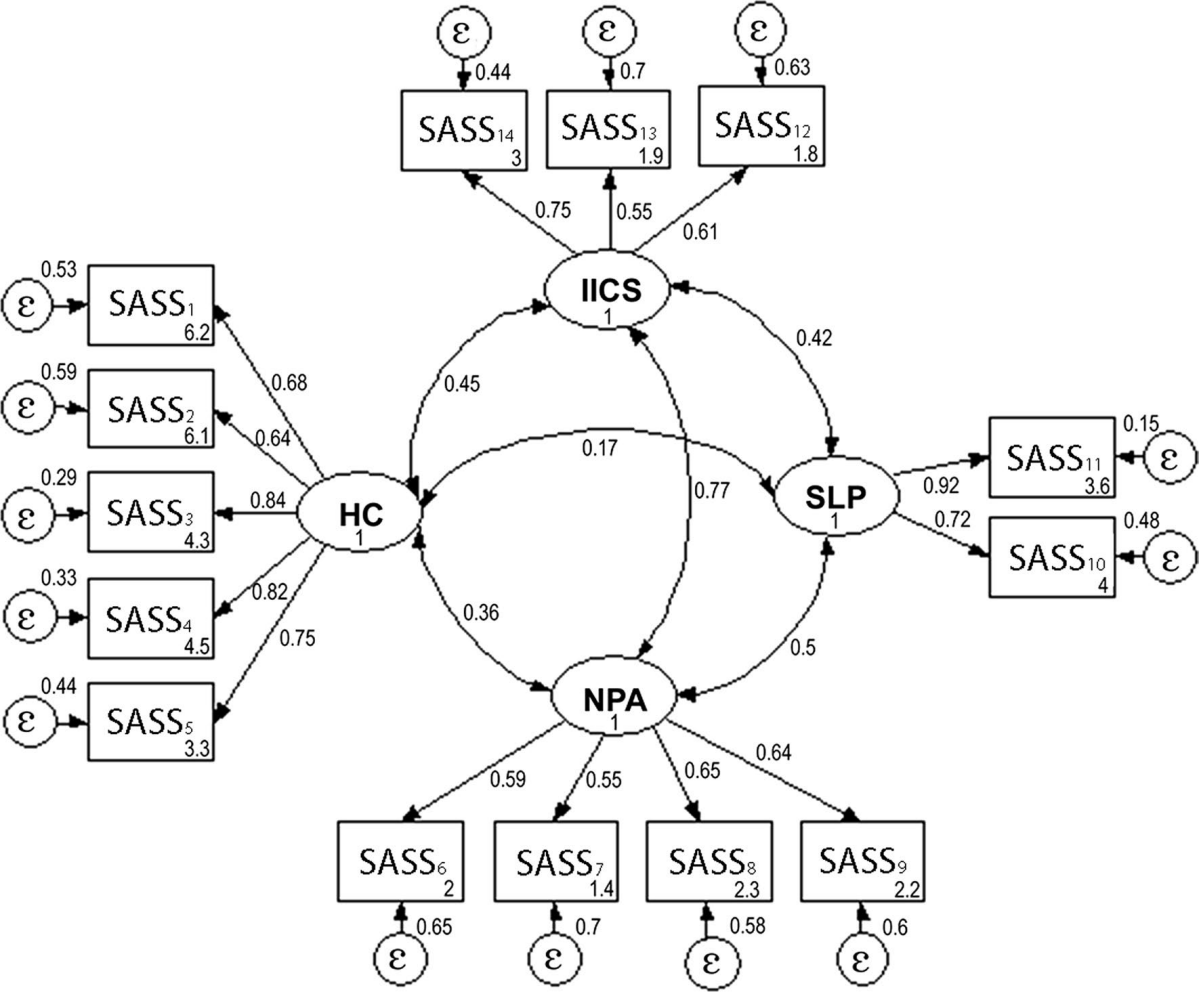
Diagram of the correlated four-factor model with standardized estimates validated in Study 2

First, a one-factor model where all items loaded on one dimension (e.g., self-care) was tested over sample 2. The chi-square was 566.17, df = 77, *p* < 0.001, CFI = 0.579, TLI = 0.503, SRMR = 0.121, BIC = 12,336.976, AIC = 12,187.266, RMSEA = 0.156. Next, the correlated four-factor model derived from the exploratory analysis was tested. The chi-square was 171.674, df = 71, *p* < 0.001, CFI = 0.913, TLI = 0.89, SRMR = 0.056, BIC = 11,976.868, AIC = 11,805.771, RMSEA = 0.074. A comparison between the goodness and fitting of both models indicated that the correlated four-factor model represented a better approximation in terms of fit and goodness. Therefore, we selected this model as a valid representation of the self-care construct (Fig. 1). The mean sub-scales scores of the SASS-14 differentiated by age are detailed in Table 2, together with the size, the standard error, and the 95% confidence interval.

**Table 2:** Age-related rating anchors of the SASS-14 total and sub-scales scores in the validation sample (n = 261)

	Size	Mean	SE	95% CI	
18–28 years					
HC	56	23.46	0.71	22.03	24.90
NPA	56	13.43	0.59	12.25	14.60
SLP	56	8.75	0.34	8.06	9.44
IICS	56	11.34	0.50	10.33	12.35
SASS	56	56.98	1.62	53.74	60.22
29–39 years					
HC	55	25.26	0.56	24.24	26.49
NPA	55	14.33	0.57	13.18	15.47
SLP	55	8.80	0.40	7.99	9.61
IICS	55	10.51	0.50	9.52	10.5
SASS	55	59	1.32	56.28	61.72
40–49 years					
HC	46	25.35	0.61	24.12	26.57
NPA	46	14.80	0.64	13.52	16.08
SLP	46	9.15	0.37	8.40	9.90
IICS	46	10.96	0.59	9.78	12.13
SASS	46	60.26	1.62	56.99	63.43
50–59 years					
HC	39	26.05	0.79	24.45	27.65
NPA	39	15.62	0.70	14.20	17.03
SLP	39	9.44	0.40	8.62	10.25
IICS	39	11.33	0.57	10.18	12.49
SASS	39	62.44	1.65	59.10	65.77
> 60 years					
HC	65	25.17	0.52	24.14	26.20
NPA	65	15.30	0.51	14.29	16.33
SLP	65	9.92	0.28	9.37	10.47
IICS	65	11.46	0.43	9.60	11.32
SASS	65	60.86	1.14	58.58	63.14

*HC* health consciousness, *NPA* nutrition and physical activity, *SLP* sleep, *IICS* intra-personal and inter-personal coping skills, *SASS* Self-care Activities Screening Scale

Regarding reliability, the Cronbach’s alpha coefficient of the SASS-14 was 0.801 in the confirmatory sample, whereas the internal consistency of its dimensions were α_HA_ = 0.85, α_DPA_ = 0.61, α_SLP_ = 0.86, and α_IICS_ = 0.57. Thus, the internal consistency of the SASS-14 was good with acceptable to high (0.57–0.86) reliability in its subscales.

### Convergent validity

All the sub-scales of the SASS-14 (including the total score) were found to be significantly and positively correlated within them and also with almost all the sub-scales of the PWBS-29-except with positive relationships and autonomy- and with the total scores of the PWBS-29 and PSS-10, supporting convergent validity between self-care and well-being and perceived stress measures (see Table 3).

**Table 3:** Pair-wise correlation coefficients between age, sex, education level, income level, self-care activities, stress and well-being (n = 261)

	Age	Sex	Edu	Inc	HC	NPA	SLP	IICS	SASS	SA	PRO	ATM	EM	PG	PL	PWBS	PSS
Age	–	0.08	− 0.06	0.40 *	0.11	0.18	0.19	− 0.02	0.16	0.22	− 0.05	0.11	0.21	0.09	0.21	0.16	− 0.21
Sex		–	− 0.09	0.17	− 0.10	− 0.06	− 0.08	− 0.10	− 0.12	0.06	− 0.05	0.10	− 0.04	− 0.08	0.04	0.01	− 0.10
Edu			–	0.13 *	− 0.15	0.05	− 0.10	0.08	− 0.05	0.02	0.31 *	0.29 *	0.18	0.15	− 0.01	0.21	− 0.17
Inc				–	0.02	0.21	0.03	− 0.10	0.07	0.27 *	0.13	0.16	0.26 *	0.15	0.25 *	0.27 *	− 0.23 *
HC					–	0.23 *	0.34 *	0.23 *	0.69 *	0.35 *	0.02	− 0.03	0.22 *	0.25 *	0.31 *	0.22	− 0.16
HPA						–	0.36 *	0.46 *	0.76 *	0.41 *	0.13	0.12	0.32 *	0.27 *	0.38 *	0.33 *	− 0.23 *
SLP							–	0.26 *	0.63 *	0.42 *	0.08	0.12	0.32 *	0.22 *	0.42 *	0.33 *	− 0.30 *
IICS								–	0.70 *	0.34 *	0.21	0.12	0.32 *	0.28 *	0.36 *	0.34 *	− 0.17
SASS									–	0.54 *	0.15	0.10	0.42 *	0.37 *	0.52 *	0.43 *	− 0.29 *
SA										–	0.41 *	0.42 *	0.72 *	0.65 *	0.86 *	0.84 *	− 0.60 *
PRO											–	0.60 *	0.54 *	0.47 *	0.33 *	0.73 *	− 0.57 *
ATM												–	0.59 *	0.45 *	0.34 *	0.75 *	− 0.59 *
EM													–	0.61 *	0.66 *	0.87 *	− 0.68 *
PG														–	0.57 *	0.76 *	− 0.52 *
PL															–	0.78 *	− 0.53 *
PWBS																–	− 0.74 *

*edu* education level, *inc* income level, *SASS* self-care activities screening scale, *PWBS* psychological Well-being Scale, *HC* health consciousness, *NPA* nutrition and physical activity, *SLP* sleep, *IICS* intra-personal and inter-personal coping strategies, *SA* self-acceptance, *PRO* positive relationships with others, *ATM* autonomy, *EM* environmental mastery, *PG* personal growth, *PL* purpose in life, *PSS* Perceived Stress Scale

*Significant correlation (*p* < 0.05, corrected for multiple comparisons with Bonferoni’s method)

Within the SASS-14 sub-scales, the highest correlation was found between NPA and IICS (r = 0.46, *p* < 0.001) and the lowest correlation was found between HC and NPA (r = 0.23, p = 0.001) and also between HC and IICS (r = 0.23, p = 0.001). The highest correlations between the SASS-14 and the PWBS-29 were found between SLP and SA (r = 0.42, *p* < 0.001) and also between SLP and PL (r = 0.42, *p* < 0.001). The correlation coefficient between the total scores of the SASS-14 with the PWBS-29 was 0.43 (*p* < 0.001) and with the PSS-10 was − 0.29 (*p* < 0.001). Therefore, this analysis confirmed convergent validity between self-care and well-being scores, and also between self-care and stress on this sample. Finally, the Cronbach’s alpha coefficients of the PWBS-29 and PSS-10 scales were 0.90 and 0.85, respectively.

## Discussion

In this study, we develop and validate a brief tool for screening self-care in general population during the COVID-19 lockdown: the SASS-14. First, an EFA suggested that the instrument has a correlated four-factor structure, interpreted as health consciousness, nutrition and physical activity, sleep, and interpersonal and intrapersonal coping strategies. This factor structure was confirmed by the CFA performed on an independent sample. Lastly, the tool demonstrated to be a reliable measure with good internal consistency and convergent validity with psychological well-being and with perceived stress measures.

Despite self-care conceptual model highlights the importance of risk avoidance (substance use or attitude and information consumption), hygiene routines (level of sunlight and differential spaces to work and rest), or social support (people to talk with or community social resources), items related to these dimensions were not represented by any stable factor in the EFA. This may be explained by the fact that some of these items can respond to activities that during the lockdown are not presenting any coherent pattern in people’s answers, as social interactions that underline some of these behaviours are very influenced by this situation (e.g. social activities, spare time or substance abuse). Moreover, it may also explain why some of these items ended included in the new dimension interpersonal and intrapersonal copying strategies, since it included activities related to introspection, social interaction or community participation that are being of help for people to overcome a stressful situation (e.g. items 12, 13 and 14). Therefore, while the self-care’s dimensions discarded by the EFA could play a relevant role in self-care, they could be more linked to contextual factors (i.e., external resources at home, community or healthcare settings) and self-care behaviours (i.e., principles and actions that support and motivate individuals to achieve the sustained adoption of health-seeking behaviours and lifestyles choices); strategies which may be inhibited during a confinement situation. Therefore, these dimensions could fit better with the second and third dimension of the Self-Care Matrix [3] and hence, evaluated separately.

There are different instruments that have been developed to evaluate self-care [25]. Authors such as [16],^18^ have reported health consciousness as a fundamental aspect in the active search for information related to self-care improvement and action. Therefore, the inclusion of this factor can be considered as a catalyst for all self-care activities. However, previous self-care instruments do not consider this factor within self-care construct, which can be considered as strength of this study. Additionally, none self-care instruments were identified to screen self-care activities that can be applied during a stressful situation as a confinement. These results suggest that, during a lockdown, people could reduce their self-care routines in order to satisfy their very basic needs. Which could imply that health prevention strategies in this kind of situations should primarily screen these basic aspects of self-care: physical, nutritional and sleep, emotional and social coping.

As expected, a significant positive correlation was found between the total score of the SASS-14 and that of the PWBS-29, indicating that the higher self-care’s scores, the higher levels of well-being. On the other hand, a significant negative correlation was found between the total score of the SASS-14 and that of the PSS-10, indicating that, the higher scores in self-care, the lower levels in perceived stress. Both results are in line with previous studies. Regarding, self-care and well-being, it has been found that performing self-care activities is associated to the health consciousness concept. Thus, people who engage with self-care activities have the willing to promote health and wellness [13, 14]. In such a way, self-care is predictive of well-being, what may be determinant of wellness participation [15]. Regarding stress, the existent literature has shown an inverse relationship between self-care and stress. A research conducted on graduate students demonstrated that daily habits related to sleep and exercise were related to a lower stress perception [48]. Likewise, self-care practices have been found to be associated to lower scores of perceived stress [49]. As for the relationship between stress and well-being, managing stress has been found to help increasing in one’s personal and professional sense of wellness and well-being [50]. In the same way, the literature has shown that less perceived stress is related to higher satisfaction with life and happiness [51].

It is also worthy to mentioned that a good educational level and high incomes indicated greater levels of well-being and lower stress perception. These results are in line with those related to the social determinants of health and thus, improving these structural factors will have a significant impact on people’s perception of stress, psychological well-being and potentially on their resources to get involved in self-care activities [52].

Nevertheless, this study is limited by several factors. First, despite the heterogeneity of Colombian samples, it may not be representative of the general population from different countries who are being affected differently by COVID-19 pandemic. These results should be replicated in other countries with more heterogeneous samples. Secondly, the temporal stability of the SASS-14 was not assessed in this study. Thus, it would be useful to evaluate the temporal consistency of the scale in future interventions. Third, the weight of some items loading in factors 2 and 4 of the SASS-14 (see Table 1) could be considered low or moderated (i.e., 0.50 and 0.51). However, it has been noted in recent similar studies that it is common in social science research to consider lower weights explained as satisfactory [47]. In this instance, decisions regarding items to retain should also include considerations of theoretical content.

Despite these limitations, it is worthy to mention some important strengths and clinical implications for the tool SASS-14. Firstly, screening self-care activities in general people could help to address future deeper evaluations and conduct interventions more targeted to specific groups who can be at risk to develop unhealthy behaviours, and hence, their health status. Research on self-care suggests that people can delay or prevent many health problems related to stress exposition, in which an unhealthy lifestyle or lack of self-care is well established as a key causative agent [1]. Likewise, The SASS-14 can be used as a short screen tool to explore self-care activities during lockdown experiences in order to prevent future health complications and identify those who would benefit most from receiving supplemental physical and psychological support during this complex situation. The SASS-14 is brief, quick and available online to complete, meaning healthcare professionals could easily administer it remotely to screen general and clinical population’s healthy routines during confinement. Furthermore, future research could also assess whether the SASS-14 is associated with later health behaviours as this would highlight the importance of using this tool to screen self-care as an important prerequisite to engage in healthy lifestyle choices. Moreover, measures related to interconnected elements of self-care, such as, self-care agency and self-care efficacy should be evaluated in combination with SASS-14 to ensure a good comprehension of the self-care process.

## Conclusions

The SASS-14 is a short, reliable tool which appears to validly measure self-care activities in Spanish-speaker general population during a confinement situation. Clinically, this tool could be especially useful for exploring quickly promoting health behaviours in general population. Likewise, this tool can be also very helpful to screen self-care during stressful experiences as confinement situations can result. Further research should aim to replicate these results to understand if this construct can be reliably measured in other countries.

## Data Availability

The data that support the findings of this study are available on request from the corresponding author. The data are not publicly available due to privacy or ethical restrictions.

## Abbreviations

SASS: Self-care Activities Screening Scale
EFA: Exploratory Factor Analysis
CFA: Confirmatory Factor Analysis
HC: Health Consciousness
NPA: Nutrition and Physical Activity
SLP: Sleep
IICS: Interpersonal and intrapersonal coping strategies
PWBS: Physichological Well-Being Scale
SA: Self-Acceptance
PRO: Positive Relationships to Others
ATM: Autonomy
EM: Environmental Mastery
PL: Purpose in Life
PG: Personal Growth
PSS: Perceived Stress Scale
